# LOXL1 confers antiapoptosis and promotes gliomagenesis through stabilizing BAG2

**DOI:** 10.1038/s41418-020-0558-4

**Published:** 2020-05-18

**Authors:** Hua Yu, Jun Ding, Hongwen Zhu, Yao Jing, Hu Zhou, Hengli Tian, Ke Tang, Gang Wang, Xiongjun Wang

**Affiliations:** 1 Precise Genome Engineering Center, School of Life Sciences, Guangzhou University, Guangzhou, 510006 China; 2 CAS Key Laboratory of Tissue Microenvironment and Tumor, Institute of Health Sciences, Chinese Academy of Sciences, University of Chinese Academy of Sciences, Shanghai, 200031 China; 3 Department of Neurosurgery, Shanghai Jiao Tong University Affiliated Sixth People’s Hospital, 600 Yishan Road, Xuhui District, Shanghai, 200233 China; 4 Department of Analytical Chemistry and CAS Key Laboratory of Receptor Research, Shanghai Institute of Materia Medica, Chinese Academy of Sciences, 555 Zuchongzhi Road, Shanghai, 201203 China

**Keywords:** CNS cancer, CNS cancer

## Abstract

The lysyl oxidase (LOX) family is closely related to the progression of glioma. To ensure the clinical significance of LOX family in glioma, The Cancer Genome Atlas (TCGA) database was mined and the analysis indicated that higher LOXL1 expression was correlated with more malignant glioma progression. The functions of LOXL1 in promoting glioma cell survival and inhibiting apoptosis were studied by gain- and loss-of-function experiments in cells and animals. LOXL1 was found to exhibit antiapoptotic activity by interacting with multiple antiapoptosis modulators, especially BAG family molecular chaperone regulator 2 (BAG2). LOXL1-D515 interacted with BAG2-K186 through a hydrogen bond, and its lysyl oxidase activity prevented BAG2 degradation by competing with K186 ubiquitylation. Then, we discovered that LOXL1 expression was specifically upregulated through the VEGFR-Src-CEBPA axis. Clinically, the patients with higher LOXL1 levels in their blood had much more abundant BAG2 protein levels in glioma tissues. Conclusively, LOXL1 functions as an important mediator that increases the antiapoptotic capacity of tumor cells, and approaches targeting LOXL1 represent a potential strategy for treating glioma. In addition, blood LOXL1 levels can be used as a biomarker to monitor glioma progression.

## Introduction

Gliomas represent approximately 70% of malignant primary brain tumors in adults and are characterized by high recurrence and low five-year survival rate [1]. Due to the existence of the blood–brain barrier, current efficient clinical treatments for glioma are almost limited to temozolomide (TMZ) chemotherapy and ionizing radiation (IR) [2]. Glioma cells can also acquire resistance to apoptosis, making the current treatments ineffective. Thus, targeting antiapoptotic factors might be a good solution to improve patient survival.

LOXs are copper-dependent monoamine oxidases that are involved in the early stage of collagen and elastin polymerization in the extracellular matrix (ECM) and in later collagen-elastin crosslinking, thereby increasing the stability of ECM [3]. The LOX family consists of LOX and LOX-like (LOXL) proteins, including LOXL1, LOXL2, LOXL3 and LOXL4 [4]. The carboxyl termini of LOX family proteins are highly conserved with a catalytic domain of 205 amino acids shared by LOXL1-4, while the amino termini of LOXL1-4 differ substantially, determining the various biological functions of these four LOXLs [5]. In general, LOXs have many important biological functions, including the regulation of cell differentiation, mobility, migration and gene expression [6]. Recent studies have shown that LOXs expressions are increased under hypoxic conditions in malignant tumors, including esophageal cancer, colorectal tissue cancer, bladder cancer and head and neck cancer, and that they function in promoting tumor metastasis [7–9]. In other types of tumors, such as prostate, liver, lung, breast and stomach cancers, LOXs are involved in inhibiting tumor proliferation [10–12]. There are also a few reports about the role of LOX family in glioma. For example, the polymorphisms in *LOX* gene, 22 G/C and 473 G/A, were associated with increased susceptibility to glioma [13]. LOX expression was regulated by IDH1 status in astrocytomas [14]. And active LOX would modulate migration by association with FAK/paxillin in invasive astrocytes [15]. More importantly, recent research discovered a symbiotic glioma-macrophage interplay, which could be considered as a novel target for PTEN-deficient glioma [16]. Also, LOXL1 could play an aggressive role in glioma through its antiapoptotic capacity via Wnt/beta-catenin signaling [17]. In addition, the lncRNA, LOXL1-AS, was required for maintaining mesenchymal characteristics of glioblastoma via NF-κB pathway [18]. However, it is still elusive how LOXL1 is upregulated and exerts antiapoptotic function by directly forming an axis with other proteins during glioma progression.

In the present study, we found that LOXL1 was involved in gliomagenesis and LOXL1 expression was specifically upregulated through the VEGFR-Src-CEBPA axis. Growth factor receptor signaling stimulation results in the activation of transcriptional programs required for survival, proliferation, invasion, and angiogenesis [19, 20]. Forced expression of LOXL1 significantly increased the antiapoptotic capacity of U87 cells, while LOXL1 depletion in LN18 or GSC11 cells heavily impaired the cell survival rate under suspended culture conditions. Protein-protein interaction network analysis indicated that LOXL1 could interact with various important proteins, especially BAG2. BAG2 is a cochaperone and well-known protein that antagonizes apoptosis. It has been reported that BAG2 expression is increased in proteasome inhibitor-induced apoptosis [21]. Additionally, BAG2 plays a pro-oncogenic role in triple-negative breast cancer cells through its antiapoptotic activity [22]. In our study, LOXL1 stabilized BAG2 by blocking K186 ubiquitination, rendering glioma cells resistant to apoptosis in nonadherent conditions. Our data suggest that LOXL1 can be a potential biomarker for guiding the clinical treatment of glioma, and the development of new drugs targeting LOXL1 may improve the curative efficacy and prolong survival in glioma patients.

## Materials and methods

### Cell lines, antibodies and reagents

U87, LN18 and 293FT cell lines were purchased from American Type Culture Collection (ATCC, USA) or obtained from the Cell Bank of Type Culture Collection of Chinese Academy of Sciences China (Shanghai, China). GSC11 cell was a kind gift from Dr. Weiwei Yang laboratory (Shanghai Institute of Biochemistry and Cell Biology, Chinese Academy of Sciences, University of Chinese Academy of Sciences, Shanghai 200031, China). Cells were tested for mycoplasma contamination and authenticated using the short tandem repeat (STR) method in the previous report [23].

Antibodies against LOXL1 (H00004016-D01P), LOXL4 (NBP2-32692), BAG2 (AF800) were purchased from Novus Biologicals (USA) for western blot. Antibodies against IRF-1(#8478), CEBPA (#8178), cMyc (ab56), GATA1 (ab181544) and AP1 (ab31419) were purchased from cell signaling technology (USA) or Abcam (UK) for chromatin IP.

EGFRi, Afatinib (BIBW2992, S1011); VEGFRi, Axitinib (S1005); Notchi, RO4929097(S1575); PI3Ki, LY294002(S1105); SMOi, Smoothened Agonist (SAG) HCl, (S7779); Srci, Bosutinib (SKI-606, S1014); PKCi, Go 6983(S2911); Akti, MK-2206 2HCl (S1078); ERKi, LY3214996 (S8534); p38i, SB203580(S1076); DKK1 (Millipore, GF170).

### Plasmids, lentivirus packaging and infection

The LOXL1-overexpressing plasmid was constructed by cloning LOXL1 from a LN18 cell line cDNA library using KOD FX Neo (TOYOBO) DNA polymerase and then subcloned into the pCDH-3′SFB vector to generate Flag-LOXL1. LOXL1 and LOXL4 knockdown plasmids were constructed by introducing annealing small hairpin RNA (shRNA) into the pGIPZ vector. The pGIPZ control vector was generated with the control oligonucleotide 5′-CTCGCTTGGGCGAGAGTAA-3′. The pGIPZ-LOXL1-shRNA plasmid was generated with shLOXL1#1: 5′-cctgggaactacatcctca-3′ oligonucleotide or shLOXL1#2: 5′-gcattaaagcagcgtatc-3′ oligonucleotide targeting the coding region of LOXL1. The target sequences used to construct pGIPZ-LOXL4-shRNA are described in the previous reports [24, 25]. The siRNAs targeting human BAG2, SRC and CEBPA were purchased from GenePharma (Shanghai, China).

Lentiviruses were amplified using standard methods in sub-confluent HEK293FT cells. GBM cell lines were infected with the lentiviruses in the presence of polybrene (Sigma) at a final concentration of 8 µg/ml. Cells were incubated with the lentivirus mixture for 72 h, digested with trypsin for passage into fresh growth medium, and then sorted based on green fluorescence to determine stable expression or knockdown. The constructed stable cell lines were amplified and used in the subsequent experiments.

### The Cancer Genome Atlas (TCGA) glioma patients’ survival analysis

TCGA glioma patients’ survival data for LOX, LOXL1, LOXL2, LOXL3 and LOXL4 were downloaded from www.tcgaportal.org. Patients were divided into two groups, low and high, according to the expression level using the best cutoff. Kaplan–Meier survival curves were reconstructed using R. The log-rank p value was reported.

### Cell culture and transfection

Cells were seeded in 35, 60, or 100 mm plates and then transfected with the indicated plasmids using PolyJet (SignaGen Laboratories) according to the manufacturer’s instructions.

### Colony formation assay

For colony formation assays, 5 × 10^3^ cells per well were plated in six-well plate in triplicate and cultured for 14 days before staining viable colonies with nitro blue tetrazolium (Sigma).

### Quantitative PCR

Total RNA was prepared from the cell samples using Trizol (Invitrogen) according to the manufacturer’s protocol. Reverse transcriptase PCR was performed using a M-MLV reverse transcriptase (Promega). Real-time PCR reactions were performed using SYBR® Premix Ex Taq (Takara) and 300 nmol/l of each primer. Amplification was performed according to the manufacturer’s protocol of the 7500 Fast Real-Time PCR Systems (Applied Biosystems).

### Cell proliferation and survival assay

Cell viability under different treatment conditions was measured using Cell Counting Kit-8 (CCK-8, Dojindo Laboratories) according to the manufacturer’s instructions. The cells were plated at a density of 10^4^ cells/well in a volume of 300 μl with triplicates. On the following day, 30 μl of the CCK-8 cell-counting solution was added to each well and incubated at 37 °C for 3 h. The absorbance of the solution was read spectrophoto metrically at 450 nm with a reference at 650 nm using a microtiter plate reader (Becton-Dickinson).

### 3D suspension culture

A total of 5 × 10^5^ cells were suspended for 48 h in a 3D Insert PS scaffold for 6-well plates, which was purchased from Sigma (Z687545). After culture for 48 h, we collected the suspended cells for western blot or apoptosis analysis.

### FACS analysis of apoptosis

Flow cytometry analyses for cell death were detected with an annexin V-fluorescein isothiocyanate (FITC)/PI kit as described previously [25]. After treatment, cells were trypsinized, collected by centrifugation, washed with PBS, and resuspended at a density of 5 × 10^5^ cell/ml with 1× annexin V binding buffer. Then 5 μl annexin V-FITC conjugates and 5 μl PI solution were added and incubated for 15 min in the dark. Finally, cells were incubated with 1× annexin V binding buffer and analyzed within 1 h by flow cytometric analysis (BD FACS Aria SORP, USA). At least 3 × 10^4^ cells were analyzed to determine the percentage of apoptotic cells.

### IP-MS and bioinformatic analysis

Mass spectrometry data were analyzed using MaxQuant 1.6.2.3 software and searched against the human Swiss-Prot database (20231 protein sequences, downloaded in December 2017) [26]. Carbamidomethyl cysteine was searched as a fixed modification, and oxidized methionine and protein N-term acetylation were set as variable modifications. Enzyme specificity was set to trypsin/P. Two missing cleavage sites were allowed. The minimum peptide length was set to 7 residues. The tolerances of first search and main search for peptides were set to 20 and 4.5 ppm, respectively. The peptide and protein false discovery rates (FDRs) were fixed at a significance level that did not exceed 0.01. The “match between runs” function was chosen with a matching time window of 1 min and alignment time window of 20 min. The protein intensity was determined using the iBAQ (intensity-based absolute quantification) method in MaxQuant.

A total of 1349 protein groups were identified after removing “reverse”, “potential contaminant”, and “only identified by site” proteins. Because this analysis was a discovery-based experiment to identify LOXL1-interacting proteins, only proteins with quantified values in two LOXL1 replicates were retained, resulting in 1176 proteins. The missing intensity values in IgG immunoprecipitates were imputed by representative noise values and the ratios were converted using a log2 transformation to obtain all protein ratios for IPs with the LOXL1 antibody to IgG. Totally, 131 proteins with a log2 (ratio) cutoff >1 were determined to be potential LOXL1 interactors.

The STRING database was used to predict protein-protein interactions. A low confidence score (0.15) was chosen to identify potential interactions. Based on the results of the enrichment analysis using STRING, proteins involved in “negative regulation of apoptotic process”, “microtubule cytoskeleton organization”, “unfolded protein response/protein folding”, “ER to Golgi vesicle-mediated transport” and “regulation of protein kinase activity” were highly represented. The interactions of these proteins were released and reconstructed into a network using Cytoscape 3.7.1 software. The gray lines indicate the relationships among these proteins and the black lines indicate the direct interactions between LOXL1 and other proteins revealed by the database.

### Intracranial injection, bioluminescence imaging, and Hematoxylin & Eosin staining

Approximately 2 × 10^5^ U87-LOXL1/U87-Vec cells, LN18-shLOXL1/LN18-NT cells or GSC11-shLOXL1/GSC11-shNT cells expressing luciferase (in 5 μl of DMEM per mouse) were injected intracranially into randomly selected 8-week-old female athymic nude mice. Mice were used in accordance with ethical regulations and the protocol was approved by the Institutional Review Board at the Institute of Health Sciences and Guangzhou university. The cell suspension was injected slowly into the mouse brain, and when tumor formation was established, further experiments based on specific needs were performed.

Briefly, a small hand-controlled twist drill with a 1 mm diameter was used to create a hole in the animal’s skull. The cell suspension was drawn up into the cuffed Hamilton syringe. The needle of the Hamilton syringe was slowly lowered into the central hole of the guide screw until the cuff rested on the screw surface.

Four mice were included in each group in each experiment after inoculation; the mice were intraperitoneally injected with 100 μl of 7.5 mg/ml D-luciferin (Xenogen) and subsequently anesthetized with isoflurane inhalation. Bioluminescence imaging with a CCD camera (IVIS, Xenogen) was initiated 10 min after the injection. Bioluminescence from the region of interest was defined manually. Background was defined using a region of interest from a mouse that was not administered an intraperitoneal injection of D-luciferin. All bioluminescence data were collected and analyzed using IVIS software. After bioluminescence imaging, animals were sacrificed, and the brain of each mouse was harvested, fixed with 4% formaldehyde, and embedded in paraffin. Tumor formation and phenotypes were determined by performing histological analysis of H&E-stained sections. The equation used to calculate the tumor volume was *V* = *ab*^2^/2. No animals were excluded from the analysis. Data represent the means ± SD of four mice.

In Fig. 2, ~1 × 10^5^ (in 5 μl of DMEM per mouse) U87 cells expressing luciferase along with reconstituted expression of Vec or LOXL1 were intracranially injected into randomized 8-week-old female athymic nude mice and then subjected to IR (γ) radiation (4 Gy) 45 or 33 days after the inoculation with the similar luciferase intensity. For a comparison of the effect of IR radiation on tumor formation, we selected animals with similar luciferase intensity from the Vec and LOXL1 groups. Four mice in each group were included. After inoculation, bioluminescence imaging of mice was performed as described above. Survival durations of the tumor-implanted mice were compared.

### Chromatin immunoprecipitation (ChIP)

Rabbit monoclonal anti-IRF-1 antibody (1:500), anti-CEBPA (1:300), rabbit monoclonal anti- cMyc antibody (1:500), anti-GATA1 antibody (1:200) and anti-AP1 (1:500) were used in ChIP assays with a rabbit monoclonal IgG (1:500; Cell signaling) as a negative control. The presence of predicted transcription factor binding regions pulled by this corresponding antibody was assessed by PCR. A small amount of pre-cleared DNA (before addition of antibodies) was set as an input control.

### CRISPR-Cas9-mediated LOXL1 gene editing through homology-directed repair

The following two pairs of LOXL1 sgRNAs near the D515 residue were designed:

sgRNA-1-S, 5′-CACCCTGCTATGACACCTACAATG-3′

sgRNA-1-A, 5′-AAACCATTGTAGGTGTCATAGCAG-3′

sgRNA-2-S, 5′-CACCATGCGGACATCGACTGCCAG-3′

sgRNA-2-A, 5′-AAACCTGGCAGTCGATGTCCGCAT-3′.

The underlined sections mark the BbsI digestion site. sgRNAs were synthesized, annealed, and ligated to the pX458 plasmid (Addgene, #48138). Construction and verification were performed according to the previously established protocols [27, 28].

### Patient specimens and immunohistochemistry (IHC) analysis

All clinical samples were approved and received from routine processes. The ethics statement of this paper was approved by the Institutional Review Board of Shanghai Sixth People’s Hospital School of Medicine. Tissue sections from paraffin-embedded human GBM and astrocytoma specimens were stained with the indicated antibodies. We quantitatively scored the tissue sections according to the percentage of positive cells and staining intensity. We rated the intensity of staining on a scale of 0–3 points: 0, negative; 1, weak; 2, moderate; and 3, strong. We assigned the following proportion scores: X indicates that *X*% of the tumor cells were stained (0 ≤ *X* ≤ 100). The score (H-score) was obtained using the formula: 3 × percentage of strongly staining area + 2 × percentage of moderately staining area + 1 × percentage of weakly staining area, with a range of 0–300 points. Scores were compared with the overall survival, which was defined as the time from the date of diagnosis to death or last known follow-up date.

### Statistical analysis

Data are presented as individual data points and as means ± SD as indicated in figure legends. Sample number (n) represents the number of independent biological samples in each experiment. Sample sizes were estimated from pilot experiments. Statistical analysis and graph creation were performed using GraphPad Prism 7.00. Unpaired *t* test, paired *t* test (two-tailed) and Pearson’s correlation test were used to determine the statistical differences. A *P* value of < 0.05 is considered to be significant. (*p < 0.05, **p < 0.01, and ***p < 0.001).

## Results

### The LOX family significantly correlates with glioma progression and displays antiapoptotic activity

**Fig. 1:**
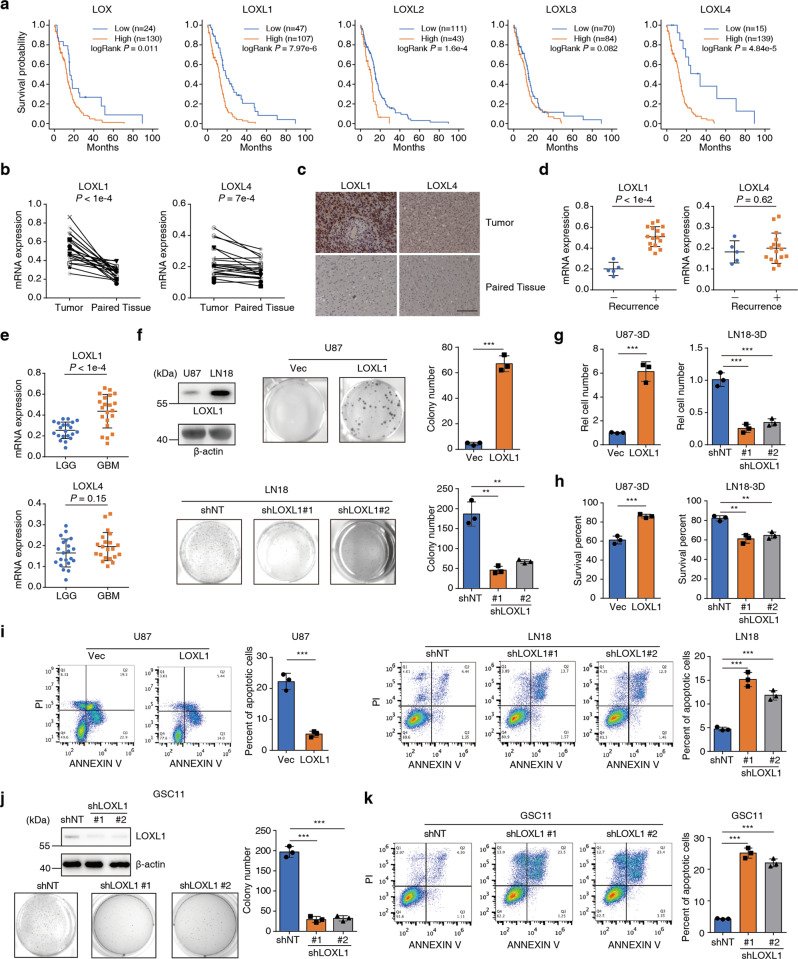
LOX family members significantly correlate with gliomagenesis and inhibit glioma cell apoptosis. **a** Kaplan–Meier curves showing differences in the overall survival of patients stratified according to their mRNA expression levels of LOX family members using the best expression cutoff (*n* = 154, log-rank test). **b** LOXL1 and LOXL4 mRNA expression levels in tumors and paired tissues (*n* = 21, paired *t* test, two-tailed). **c** IHC staining of the LOXL1 and LOXL4 proteins in tumor and paired tissues (Scale bar, 100 µm). **d** LOXL1 and LOXL4 mRNA expression in tissues from patients with or without recurrence. 21 glioma samples were divided into two groups: recurrence (+, *n* = 16) or nonrecurrence (-, *n* = 5) (means ± SD, unpaired *t* test, two-tailed). **e** LOXL1 and LOXL4 mRNA expression in tissues from patients with LGG and GBM. LGG: low grade glioma (LGG, *n* = 21; GBM, *n* = 21; means ± SD, unpaired *t* test, two-tailed). **f** Colony formation assays of U87-Vec, U87-LOXL1, LN18-shNT and LN18-shLOXL1 cells (the data are presented means ± SD, unpaired *t* test, two-tailed). **g**–**i** The proliferation and percentages of surviving and apoptotic U87-Vec, U87-LOXL1, LN18-shNT and LN18-shLOXL1 cells cultured under 3D conditions were examined (means ± SD, unpaired *t* test, two-tailed). **j**, **k** Colony formation and apoptotic assay were performed after depletion of LOXL1 in GSC11 cells.

To discover the role of the LOX family in glioma progression, we compiled the expression data of LOX family genes and determined their correlation with the survival time of glioma patients in the TCGA database. We found that patients with the low expression of LOX family coding genes, especially LOXL1 and LOXL4, survived longer than those with high expression of LOXL1 and LOXL4, using the best expression cutoff (Fig. 1a). Meanwhile, we mined the clinical significance of all the LOX family coding genes by expanding samples from glioma to other types of tumors. As shown in Supplementary Fig. 1a, left panel, LOXL1 specially implicates glioma but no other tumors. Of note, LOXL2 expression is probably involved in more types of cancer, which is consistent with the previous report [29]. In Supplementary Fig. 1a, right panel, the prognostic analysis of LOXL1 in glioma was performed using another four datasets from R2 Genomics Analysis and Visualization Platform. All data suggested the significant correlation of LOXL1 elevation to worse prognosis. Further, we provided the data analysis of our own clinical tumor cohorts (*n* = 80) and multivariate analysis after controlling for age, gender and IDH mutation. Our own data also revealed the correlation of higher expression of LOXL1 to worse prognosis (Supplementary Fig. 1b). We analyzed clinical samples to detect the expression levels of LOXL1 and LOXL4 in both tumor and matched adjacent tissues. The results showed that LOXL1 and LOXL4, particularly LOXL1, were expressed at higher levels in tumor tissues (Fig. 1b). In tumors, IHC result showed that LOXL1 and LOXL4 proteins exhibited a dense distribution, especially LOXL1 (Fig. 1c). The invasiveness and recurrence of glioma generally result in a high mortality rate [30]. Patients with recurrent glioma also showed higher LOX family gene expression, especially LOXL1 (Fig. 1d). Glioma grades are roughly divided into low grade glioma (LGG) and GBM, and LGG has the potential to progress into GBM. We also mined TCGA data for the LOX family gene expression in LGG and GBM and found similar results (Supplementary Fig. 1c). LOXL1 was expressed at a two-fold higher level in GBM than in LGG at the mRNA level, suggesting that LOXL1 may act as a risk factor for glioma progression (Fig. 1e). The data presented above imply that the expression levels of LOX family genes are closely related to the prognosis of glioma, and these genes may serve as potential markers for monitoring the progression of glioma.

As LOXL1 showed more significance in glioma progression, we tested LOXL1 expression in U87 and LN18 cells (Fig. 1f, upper panel), and then used U87 cells as the parent cell line for forced LOXL1 expression (U87-LOXL1) and LN18 cells for LOXL1 knockdown (LN18-shLOXL1). We found that U87-LOXL1 cell proliferation was reduced (Supplementary Fig. 1d) while the colony formation of the LOXL1 overexpression cells increased in number and size (Fig. 1f, upper panel). As forced expression might result in artificial results, we thus conducted the same assays in LN18-shLOXL1 cells with two distinct shRNAs. As shown in Supplementary Fig. 1e, the proliferative capacity of LN18-shLOXL1 cells was slightly increased, while the ability to form clones was significantly decreased (Fig. 1f, lower panel). We knocked down the LOXL4 gene in LN18 cells. As shown in Supplementary Fig. 1f, decreased LOXL4 expression did not affect the proliferative capacity of LN18 cells, whereas the ability to form clones was significantly decreased. For testing apoptosis, it’s difficult to collect cells from colony formation assay while we can collect cells from nonadherent cultures. Furthermore, we examined the effect of LOXL1 expression on cell survival and apoptosis in nonadherent cells. U87-LOXL1 cells are more suitable for nonadherent assays than U87 control cells, as evidenced by the increased nonadherent proliferation and cell survival. In contrast, the LN18-shLOXL1 cells showed decreased nonadherent proliferation and cell survival (Fig. 1g, h). Under nonadherent condition, we examined the cell cycle of U87-Vec, U87-LOXL1 cells, LN18-shNT and LN18-shLOXL1 cells, showing that LOXL1 promoted cell cycle progression (Supplementary Fig. 1g).

Both colony formation and nonadherent assays require cells to resist apoptosis and increase survival. Therefore, PI/Annexin V double-staining was performed to assess the ability of LOXL1 to promote glioma cell resistance to apoptosis. Upon LOXL1 overexpression, the proportion of apoptotic cells decreased, while the number of apoptotic LN18-shLOXL1 cells increased three- to four-fold compared to that of LN18-shNT cells (Fig. 1i). We used GSC11, a cancer stem cell line, to perform colony formation and apoptotic assays after depletion of LOXL1 with two distinct shRNAs and observed the consistent results with LN18 (Fig. 1j, k). These results suggest that LOXL1 promotes glioma cell survival and especially inhibits apoptosis in a nonadherent state.

### LOXL1 promotes glioma progression and enhances the resistance of tumor cells to IR

**Fig. 2:**
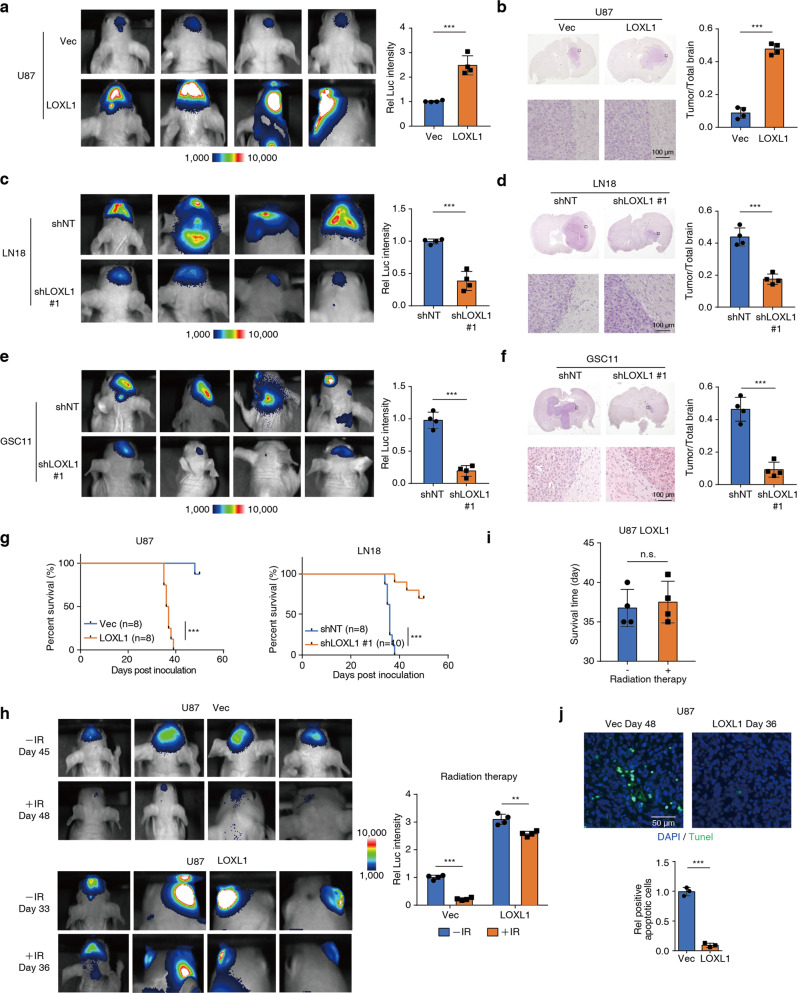
LOXL1 promotes gliomagenesis and enhances IR resistance. **a**,**b** U87 cells stably expressing luciferase were engineered to overexpress LOXL1 or Vec as a control. These cells (2 × 10^5^ per mouse) were intracranially injected into athymic nude mice. Bioluminescence imaging of tumor growth was conducted (**a**, left panel). Real-time images are presented, and the luciferase intensities were quantified (**a**, right panel). After 35 days, the tumors were removed from the mouse brains and examined. Representative images of hematoxylin and eosin (H&E)-stained coronal brain sections from tumor xenografts are shown. Scale bar, 100 μm (**b**). Representative images of tumor boundaries are presented. The data are presented as the mean luciferase intensities ± SD of 4 mice per group. ***p < 0.001. **c, d** LN18 cells stably expressing luciferase were transfected with shLOXL1 to knock down LOXL1 or shNT as a control. The processes of bioluminescence imaging and H&E staining are described in Fig. 2**a, b**. *n* = 4. **e, f** GSC11 cells stably expressing luciferase were transfected with shLOXL1 to knock down LOXL1 or shNT as a control. The processes of bioluminescence imaging and H&E staining are described in Fig. 2**a, b**. **g** Survival durations of mice injected with glioma cells (log-rank test). **h-j** Cells were intracranially injected into randomly selected athymic nude mice (four mice per group). After the U87-Vec and U87-LOXL1 groups reached appropriate tumor sizes, the mice were treated with or without IR (γ) radiation (4 Gy) and continually maintained for 3 days. Bioluminescence imaging of tumor growth was conducted. Real-time images are presented (**h**, left panel), and the luciferase intensities were quantified (**h**, right panel). The data are presented as the mean luciferase intensities ± SD of 4 mice per group. Survival durations of these implanted mice were compared (**i**). Furthermore, a TUNEL assay was performed to determine the number of apoptotic cells in the tumors following exposure to IR (**j**). ***p < 0.001. n.s., not significant.

To explore the biological function of LOXL1 in mediating antiapoptotic activity, we then examined the ability of U87-LOXL1 cells, LN18-shLOXL1 cells and GSC11-shLOXL1 cells to form solid tumors. As shown in Fig. 2a, after injection of tumor cells into the brain, mice implanted with U87-LOXL1 cells exhibited an approximately 3-fold increase in tumor formation compared with control mice. Hematoxylin & eosin (H&E) staining confirmed that all solid tumors were derived from the brain (Fig. 2b).

Next, we investigated the role of LOXL1 in brain tumor development using LN18-shLOXL1 cells or GSC11-shLOXL1 cells stably expressing luciferase. These genetically modified glioma cells were then injected intracranially into nude mice. LN18-shLOXL1 cells or GSC11-shLOXL1 cells displayed much slower tumor growth than their respective control cells (Fig. 2c, e). H&E-stained coronal brain sections consistently showed much smaller tumors in mice injected with LN18-shLOXL1 cells or GSC11-shLOXL1 cells than that in the control mice (Fig. 2d, f).

Mice injected with U87-LOXL1 cells died within 40 days, while 7 of the 8 control mice survived longer than 45 days. This finding is consistent with the TCGA data shown in Fig. 1a, showing that high LOXL1 expression is correlated with reduced survival times in patients or mice with glioma. Besides, the survival times of mice injected with LN18-shLOXL1 cells was significantly increased. Seven of the ten mice injected with LN18-shLOXL1 cells survived longer than 50 days, while all the control mice died within 38 days (Fig. 2g). Thus, LOXL1 may be specifically employed by tumor cells to resist apoptosis and may represent a therapeutic target for treating invasive brain tumors.

Due to the ability to induce apoptosis, IR has been used extensively to treat multiple types of human cancers, including glioma. We wondered whether increased LOXL1 expression was involved in the resistance of tumor cells to IR. In detail, we exposed mice (45 or 33 days after injection with U87-Vec or U87-LOXL1 cells, respectively) to IR or control treatment and found that mice injected with U87-Vec cells showed dramatically decreased tumor size, while the tumor size was barely changed in mice injected with U87-LOXL1 cells (Fig. 2h). In addition, the survival times of mice with U87-LOXL1 cells following treatment with or without IR were similar (Fig. 2i). Transferase dUTP nick end labeling (TUNEL) staining confirmed a higher percentage of apoptotic cells in brain tumors composed of U87-Vec cells than U87-LOXL1 cells (Fig. 2j). H&E staining presented a smaller reduction of tumor size in brain tumors composed of U87-LOXL1 cells than U87-Vec cells after IR treatment (Supplementary Fig. 2a). Ki67 staining showed a smaller decrease of proliferated cells in brain tumors composed of U87-LOXL1 cells than U87-Vec cells after IR treatment, indicating overexpression of LOXL1 could counteract the IR efficiency by conferring antiapoptotic activity (Supplementary Fig. 2b). Consequently, after IR treatment, the prolonged survival time of mice bearing U87-LOXL1 cells was much shorter than that of mice bearing U87-Vec cells (Supplementary Fig. 2c). These results suggest that LOXL1 promotes tumor cell survival by sequentially upregulating antiapoptotic activity.

### LOXL1 confers antiapoptotic activity by interacting with apoptosis-related modulators

**Fig. 3:**
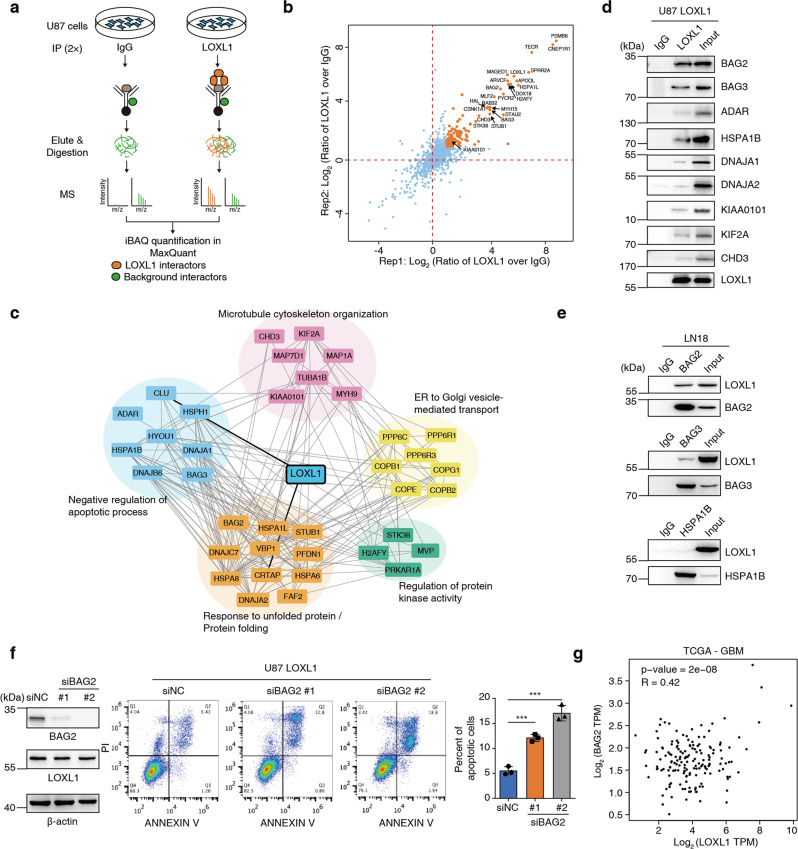
LOXL1 interacts with apoptosis-related modulators. **a** Schematic of LOXL1 interactor discovery. The potential interactors were presented at higher levels in the LOXL1 experimental group than in the IgG control group. Two replicates of IP-MS were conducted. **b** Scatter plots of the log_2_ ratios of iBAQ intensities for the proteins quantified using MS in the LOXL1 Co-IP samples from two replicates compared with the IgG control samples. Proteins displayed in red displayed a fold change of > 2 in two replicates and were determined to potentially interact with LOXL1. **c** Protein-protein interactions among potential LOXL1 interactors, as revealed by the STRING database. The gray lines indicate the relationships among these proteins, and the black lines indicate the direct interactions between LOXL1 and other proteins. **d** LOXL1 interacts with multiple proteins, especially BAG2 in U87 cells overexpressing LOXL1. **e** Endogenous reciprocal Co-IP assay was further performed to test the protein-protein interaction between LOXL1 and BAG2, BAG3 or HSPA1B in LN18 cells. **f** BAG2 was depleted by transiently transfecting specific siRNA and siNC was used as a negative control. Indicated antibodies against BAG2 and LOXL1 were used to test their protein levels. The percentages of apoptotic cells were examined. (means ± SD, unpaired *t* test, two-tailed). **g** The scatter plot of correlation data between LOXL1 and BAG2 in glioma was provided by mining TCGA database.

To clarify the mechanism underlying the antiapoptotic activity of LOXL1 in glioma cells, we suspended U87-LOXL1 cells, cultured them for 48 h, collected cells for immunoprecipitation (IP) with a LOXL1-specific antibody, separated the samples with SDS-PAGE and then analyzed by liquid chromatograph-mass spectrometer (LC-MS) (Fig. 3a and Supplementary Fig. 3a). The results showed that 131 proteins increased in abundance by more than 2-fold in the LOXL1 antibody-enriched samples compared to those in the IgG control samples (Fig. 3b). Additionally, as revealed by protein-protein interaction prediction and enrichment analyses using the STRING database [31], proteins involved in “negative regulation of apoptotic processes”, “microtubule cytoskeleton organization”, “unfolded protein response/protein folding”, “ER to Golgi vesicle-mediated transport” and “regulation of protein kinase activity” were highly increased, suggesting the potential antiapoptotic function of LOXL1 (Fig. 3c). Based on the MS analysis result, we selected 9 LOXL1 interaction candidates. We found that all 9 candidates interacted with LOXL1 in the Co-Immunoprecipitation (Co-IP) assay, with BAG2 showing as the best interactor for LOXL1 (Fig. 3d). Endogenous reciprocal Co-IP assay was further performed to test the protein-protein interactions between LOXL1 and BAG2, BAG3 or HSPA1B, revealing that BAG2 was the top interactive candidate (Fig. 3e). We then knocked down BAG2 in U87-LOXL1 cells by specific siRNAs and found that apoptotic cells considerably increased without affecting LOXL1 expression (Fig. 3f). Of note, knocking down of BAG2 reduced the invasion of LN18 and GSC11 cells (Supplementary Fig. 3b, c). The expression levels of LOXL1 and BAG2 are positively correlated (*R* = 0.42) in glioma and the correlation *R* value ranks fourth in all the 33 tumor types of TCGA (Supplementary Fig. 3d). The scatter plot of LOXL1-BAG2 in glioma was provided in Fig. 3g. Based on these results, we speculate that LOXL1 targets multiple apoptosis-related proteins, especially BAG2, to inhibit apoptosis.

### LOXL1 promotes BAG2 stability, requiring both its enzymatic activity and direct interaction with BAG2

**Fig. 4:**
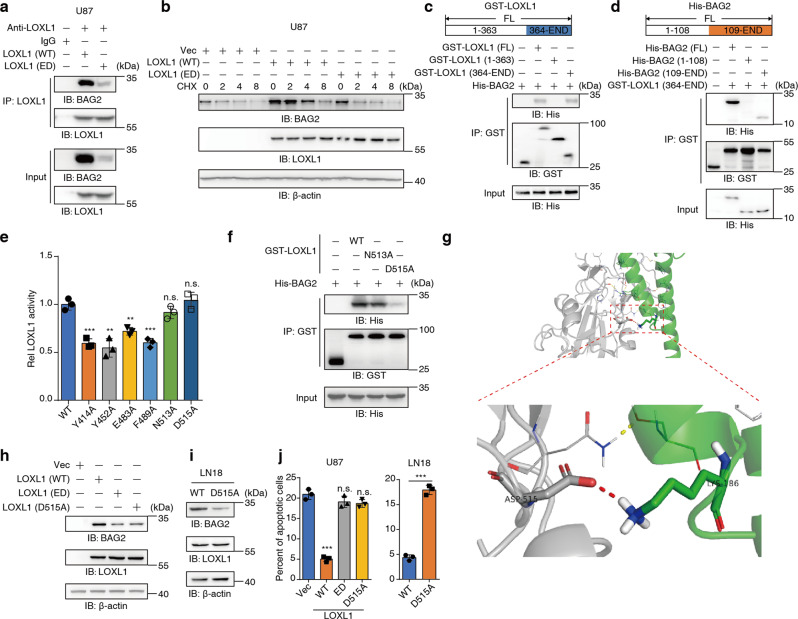
LOXL1 regulates BAG2 stability through both its enzymatic activity and direct interaction with BAG2. **a**, **b** BAG2 protein levels were increased by LOXL1. U87 cells were transiently transfected with plasmids overexpressing wild type (WT) or ED mutant LOXL1, and Vec was used as a negative control. CHX (cycloheximide, 1 μM) was used to treat cells over time (**b**). **c** GST-LOXL1, including full length (FL), 1 to 363 AAs (1-363) and 364 to the end AAs (364-END), was incubated with His-BAG2. **d** His-BAG2, including full length (FL), 1 to 108 AAs (1-108) and 109 to the end AAs (109-END), was incubated with GST-LOXL1. **e** Molecular simulations were performed to find the potential sites required for interacting with the BAG domain of BAG2. Then, measurement of LOXL1 enzymatic activity was performed. **f** D515A mutation reduced the direct interaction between LOXL1 and BAG2. GST-LOXL1 (including WT, N513A and D515A) was incubated with His-BAG2. **g** The interaction diagram of LOXL1-D515 with BAG2-K186. **h** U87 cells were transiently transfected with plasmids overexpressing WT, ED or D515A mutant LOXL1. **i** Endogenous BAG2 was determined with a specific antibody when LOXL1 D515 was mutated into A515. **j** The reduced interaction between LOXL1 and BAG2 decreased the ability of glioma cells to resist apoptosis.

To determine whether LOXL1 enzymatic activity will affect the interaction between LOXL1 and BAG2, we overexpressed wild type (WT) and enzymatically dead (ED, H449, 451, 453Q mutations) LOXL1 in U87. The results indicate that LOXL1 interacts with BAG2, and loss of the enzyme activity of LOXL1 destabilizes BAG2 (Fig. 4a). It is possible that LOXL1 overexpression may upregulate BAG2 mRNA. However, loss of LOXL1 slightly decreased BAG2 mRNA (Supplementary Fig. 4a, b). Furthermore, we observed that LOXL1 overexpression could stabilize BAG2 protein levels (Fig. 4b and Supplementary Fig. 4c).

To narrow down the interacting region between LOXL1 and BAG2, we truncated LOXL1 and BAG2 at the N terminal and C terminal separately. A GST pull-down assay showed that loss of the LOXL1 C terminal seriously impaired the interaction between LOXL1 and BAG2 (Fig. 4c). Similarly, deletion of the BAG2 C terminal reduced its direct interaction with LOXL1 (Fig. 4d). Molecular simulations preliminarily showed that Y414, Y452, E483, F489, N513 and D515 of LOXL1 could contribute to its interaction with the BAG domain of BAG2 (Supplementary Table. 1). To further determine the interacting sites in the LOXL1-C terminal and BAG2-C terminal, we mutated the above six amino acids into Ala and found that N513A and D515A had no effect on LOXL1 enzymatic activity (Fig. 4e), while D515 was more suitable than N513 for BAG2 binding (Fig. 4f). Based on the results of the molecular simulations and interaction assays, we created an interaction diagram of LOXL1-D515 with BAG2-K186 (Fig. 4g). We then wondered whether the D515A mutation affected BAG2 protein level after BAG2 disassociation from LOXL1. Upon expressing LOXL1 (D515A) in U87 cells, the interaction between LOXL1 and BAG2 was abrogated, while BAG2 protein level was similar to that in LOXL1-ED mutant U87 cells (Fig. 4h). After endogenous mutation of D515 to A515 in LN18 cells, BAG2 protein stability was also strongly impaired (Fig. 4i). In U87 or LN18 cells, reducing the interaction between BAG2 and LOXL1 resulted in the loss of apoptosis resistance, similar to that induced by the LOXL1 ED mutation (Fig. 4j). These data suggest that LOXL1 regulates BAG2 stability, requiring both its enzymatic activity and a direct interaction with BAG2.

### LOXL1 prevents BAG2 ubiquitylation at K186 through interacting with BAG2

**Fig. 5:**
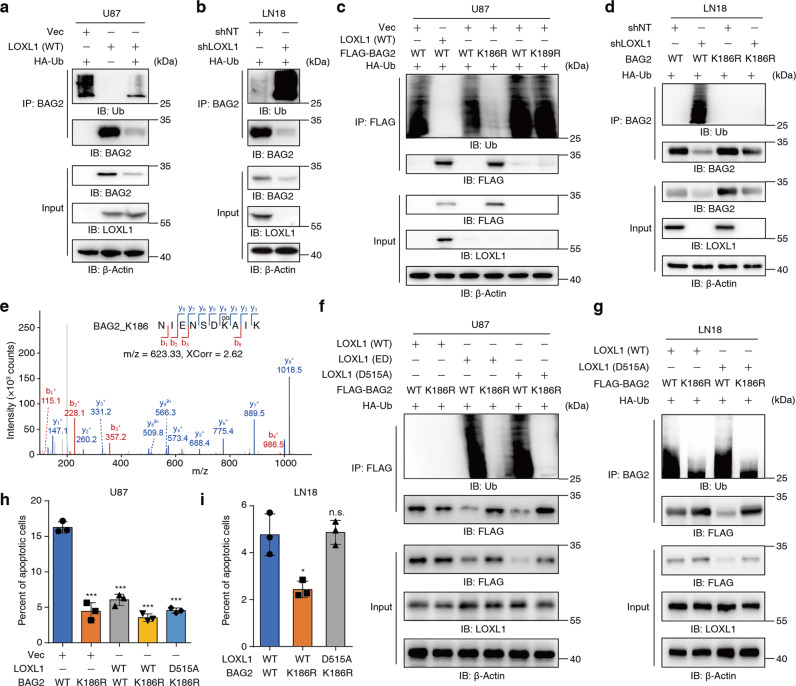
LOXL1 prevents BAG2 ubiquitylation at K186. **a** Overexpression of LOXL1 in U87 cells reduced BAG2 ubiquitylation. An antibody against BAG2 was applied to enrich the BAG2 protein levels, and BAG2 ubiquitylation was then tested. **b** Depletion of LOXL1 in LN18 cells enhances BAG2 ubiquitylation. **c** Mutation of K186 to R186 decreased BAG2 ubiquitylation. U87 cells were transiently transfected with the corresponding plasmids. BAG2 was tagged with FLAG and then immunoprecipitated with an antibody against FLAG. **d** K186 in BAG2 was mutated to R186 in LN18 cells, and BAG2 ubiquitylation was then tested. **e** MS analysis identified BAG2 K186 ubiquitylation. The spectrum of the ubiquitylation-modified K186 peptide was identified with an m/z value of 623.33 and a SEQUEST XCorr value of 2.62. **f**, **g** Both LOXL1 enzymatic activity and D515 were required for preventing the ubiquitylation of BAG2 at K186 and stabilizing the BAG2 protein. **h**, **i** Prevention of BAG2 K186 ubiquitylation enhanced the antiapoptotic activity of glioma cells.

We speculated that BAG2 stability was regulated by ubiquitin for two reasons: (1) LOXL1-D515 probably interacts with BAG2-K186, and (2) it has been well established that lysine ubiquitin modification modulates protein stability. We observed the ubiquitylation in BAG2 and LOXL1 overexpression dramatically reduced the ubiquitin modification. Both BAG2 ubiquitylation and LOXL1 depletion resulted in unstable BAG2 protein (Fig. 5a, b). We then mutated K186 to R186 or K189 to R189 and found that the K186R mutation, but not the K189R mutation, abrogated BAG2 ubiquitylation (Fig. 5c). Endogenous K186 of BAG2 was mutated to R186, resulting in BAG2 stabilization, even in the absence of LOXL1 (Fig. 5d). Consistently, we observed BAG2-K186 and BAG2-K189 ubiquitylation modifications in LN18 cells by MS analysis (Fig. 5e and Supplementary Fig. 5). As shown in Fig. 5f, both LOXL1 enzymatic activity and the LOXL1/BAG2 interaction modulated BAG2 ubiquitylation at K186 and thus protein stability. The endogenous K186R mutation of BAG2 protein also resulted in BAG2 stabilization, even when disassociated with the LOXL1-D515A mutant protein (Fig. 5g). K186R mutant cells also exhibited antiapoptotic activity, even when LOXL1 disassociated from BAG2 (Fig. 5h, i). These results suggeste that LOXL1 stabilizes BAG2 by interacting with BAG2, thus preventing ubiquitylation of BAG2 at K186. Importantly, LOXL1 enzymatic activity also contributes to the maintaining of BAG2 protein stability during this process. Considering the lysyl oxidase activity of the LOX family, we speculated that LOXL1 activity might compete for BAG2-K186 ubiquitylation by reacting with the side chain of K186.

### VEGFR-Src axis signaling increases LOXL1 expression which positively correlates with antiapoptotic gene expression in clinical samples

**Fig. 6:**
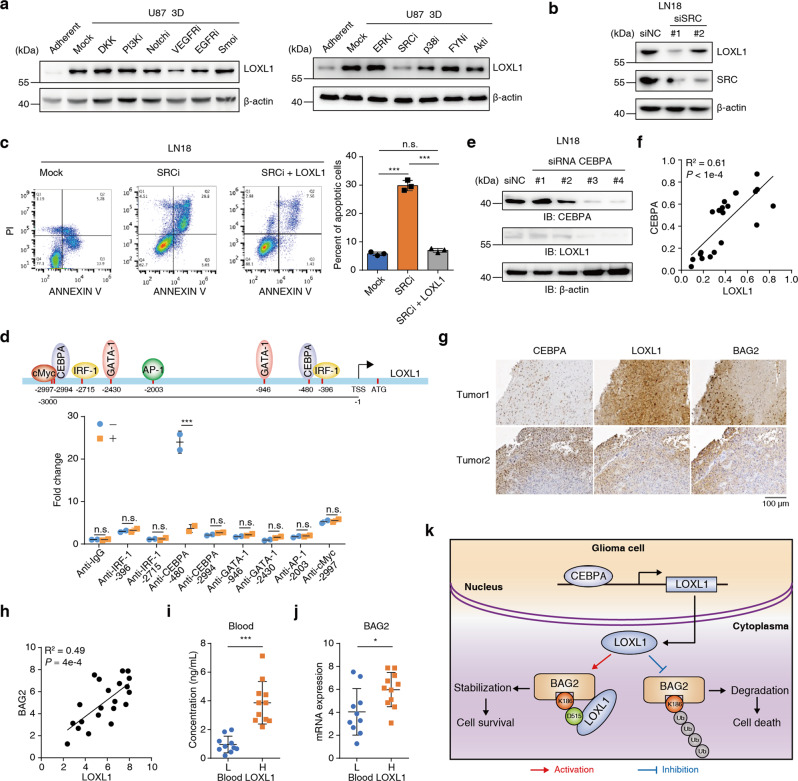
VEGFR-Src axis signaling increases LOXL1 expression which positively correlates with BAG2 during glioma progression. **a** Left panel, inhibitor screen of receptor-mediated signaling pathways required for LOXL1 upregulation in U87 cells; right panel, inhibitor screen of central kinases required for LOXL1 upregulation in U87 cells. **b** Knocking down of *SRC* gene by siRNA reduced LOXL1 protein in LN18 cells. **c** Forced expression of LOXL1 rescued the inhibition of Src kinase activity, as determined using apoptosis assays (means ± SD, one-way ANOVA). **d** Upper panel, Promo web software predicted the potential transcriptional factors that bound to the LOXL1 promoter (3000 bp upstream of the TSS); Lower panel, a ChIP assay identified that CEBPA targeted the LOXL1 promoter at 480 base pairs upstream of the TSS in LN18 cells (means ± SD, unpaired *t* test, two-tailed). **e** Knocking down CEBPA reduced LOXL1 expression in LN18 cells. Four pairs of siRNAs were applied to target the CEBPA gene. **f** Real-time qPCR analyses of glioma specimens were performed. The correlation of LOXL1 expression with CEBPA is shown as R^2^ and p value. **g** Representative images of IHC staining of glioma specimens are shown. Scale bar, 100 μm. **h** Semi-quantitative scoring (using a scale from 0 to 300 points) was conducted, and Pearson’s correlation test was performed and evaluated using the R^2^ and p value. **i** Blood LOXL1 levels were measured using ELISA, and 21 glioma specimens were divided into two groups: high (H) or low (L) (means ± SD, unpaired *t* test, two-tailed). **j** BAG2 levels were higher in the H group than in the L group. (means ± SD, unpaired *t* test, two-tailed) **k** Diagram showing the mechanism by which LOXL1 exerts its antiapoptotic activity.

The TGF-beta signaling pathway increased the expression of LOX family genes [32]. We added the inhibitor of the TGF-beta signaling pathway to the medium of adherent or nonadherent cells. As shown in Supplementary Fig. 6a, LOXL1 expression was significantly increased in nonadherent cells, and this effect was not altered by TGF-beta inhibitors. There are multiple signaling pathways involved in regulating antiapoptotic effects, and we selected inhibitors of these important signaling pathways [20]. After screening, a pan-VEGFR inhibitor effectively inhibited the upregulation of LOXL1 (Fig. 6a left panel and Supplementary Fig. 6b). The VEGFR signaling pathway has been reported to play an important role in glioma progression and chemotherapy resistance [33, 34] and employs a wide range of downstream signals, such as MAPK, Src, Akt and others [35, 36]. Therefore, we further identified the signaling molecules responsible for VEGFR signal transduction. A Src inhibitor effectively reduced the upregulation of LOXL1 in LN18 and GSC11 cells (Fig. 6a right panel and Supplementary Fig. 6c, d). Consistently, knocking down of SRC in LN18 and GSC11 cells by siRNA also inhibited LOXL1 expression (Fig. 6b and Supplementary Fig. 6e), thus demonstrating that the VEGFR-Src axis is an important pathway inducing LOXL1 expression. Following Src kinase inhibition in LN18 cells, LOXL1 expression was reduced, and cells exhibited a similar phenotype to U87 cells cultured under 3D conditions. Consequently, the Src inhibitor substantially increased the proportion of apoptotic LN18 cells, as shown in Fig. 6c. We then observed that Src kinase activity was required for upregulating LOXL1 at the transcriptional level (Supplementary Fig. 6f). We used the online software PROMO [37] to predict the transcription factor binding site within 3000 base pair (bp) from the transcription start site (TSS) of LOXL1 (Fig. 6d), and the corresponding PCR primers were designed. A ChIP assay revealed a significant decrease in the binding efficiency of CEBPA to the surrounding -480 bp region upon Src inhibitor treatment, while that of the other predicted transcription factors did not obviously change (Supplementary Fig. 6g). CEBPA was knocked down by siRNA, resulting in reduced LOXL1 expression in LN18 and GSC11 cells (Fig. 6e and Supplementary Fig. 6h). We surmise that the VEGFR-Src-CEBPA axis is important for upregulating LOXL1 expression in nonadherent cells, which in turn confers glioma cells the ability to resist apoptosis.

We measured the mRNA expression of CEBPA and LOXL1 in 21 glioma specimens to determine the clinical relevance of our finding that LOXL1 was upregulated by CEBPA. As shown in Fig. 6f, the positive correlation between CEBPA and LOXL1 was quantified (*R*^2^ = 0.61). We narrowed down 21 glioma specimens to 14 by selecting the samples with a better correlation between CEBPA and LOXL1 gene expression. Then, we assessed correlations between CEBPA, LOXL1 and BAG2 at the total protein level in these 14 samples (Supplementary Fig. 6i). Furthermore, we performed IHC analyses in these samples using antibodies against CEBPA, LOXL1 and BAG2. Representative images of CEBPA, LOXL1 and BAG2 are shown in Fig. 6g. The level of LOXL1 was positively correlated with that of BAG2 in the tissue. Quantification of the staining on a scale of 0–300 points revealed a significant positive correlation between LOXL1 and BAG2 (*R*^2^ = 0.49) (Fig. 6h).

LOXs are secreted extracellularly to remodel the ECM and are soluble in blood, suggesting that the LOXL1 level in blood may represent a potential indicator for glioma cell survival following TMZ and IR treatment. We examined LOXL1 levels in blood samples (blood LOXL1) from 21 patients with glioma and divided them into two groups according to the levels of secreted LOXL1 (Fig. 6i). Notably, patients with higher levels of LOXL1 in their blood had higher protein levels of BAG2 in their glioma tissues (Fig. 6j).

## Discussion

In this study, we found that LOX family proteins, especially LOXL1, can protect glioma cells from anoikis and IR stresses, rendering cells resistant to apoptosis. In detail, LOXL1 is upregulated by the VEGFR-Src-CEBPA axis in suspended glioma cells, and LOXL1 and BAG2 proteins can interact in glioma cells, preventing BAG2-K186 ubiquitylation depending on LOXL1 enzymatic activity and stabilizing BAG2 to ultimately promote cell survival (Fig. 6k).

Extracellular proteins are increasingly important as therapeutic targets, and multiple lines of evidence implicate the tumor microenvironment as a pivotal factor for regulating tumor initiation and progression [7, 9]. Members of the LOX family are secreted by tumors and are the subject of extensive efforts aimed at understanding their roles in cancer [6]. Recently, Chen et al reported that LOX secreted by tumor cells facilitated the interactions between symbiotic macrophage and glioma cells, which led a synthetic lethality in PTEN-null glioma and provided a new strategy for targeting glioma [16]. This suggests that we should be more cautious when using survival curves as clinical reference, especially considering the number of tumor-associated macrophages and their gene expression profilings.

Tumor cells have developed novel strategies to acquire resistance and survival [38]. When local nutrients and spaces are not sufficient or tumors face drug-related challenges, tumor cells tend to detach from their primary sites and begin to find new niches to colonize, which is known as tumor metastasis [39]. In this process, tumor cells change from a state of adhesive growth to one of suspension, leading to a type of apoptosis known as anoikis [40], which is the first-line barrier faced by metastasizing tumor cells. However, a small population of tumor cells can acquire the ability to resist anoikis, allowing the cells to adhere, proliferate and form new clones when they reach suitable sites. In this study, LOXL1 was shown to modulate the antiapoptotic process during IR stress and nonadhesive growth through a protein interaction network with antiapoptotic proteins, especially BAG2.

In addition to their roles in ECM remodeling, the LOX family also participates in regulating intracellular proteins. LOX-induced inhibition of NF-κB is mediated by its propeptide domain. Furthermore, the tumor-suppressive effects of LOX on RAS-mediated transformation are mediated by the RAF-heat shock protein 70 (HSP70) axis, which is also linked to NF-κB [41]. LOXL2 and LOXL3 have been shown to interact with and stabilize the transcription factor Snail, preventing its degradation by glycogen synthase kinase 3β (GSK3β) and leading to reduced CDH1 expression [42]. Recently, LOXL4 has been shown to promote p53 activation by interacting directly with p53 [25]. The LOX family also has intracellular enzymatic activities. LOXL3 interacts with STAT3 to promote lysine oxidation and deacetylation of its acetylated K685 residue [43]. LOXL1 activates Wnt/beta-catenin signaling to accelerate cell proliferation and cell growth in glioma [17]. However, how LOXL1 activates Wnt/beta-catenin signaling and whether LOXL1 directly interacts with the members of Wnt/beta-catenin signaling are unknown.

In the present study, we described the cancer-promoting effect of LOXL1, which directly originated from its intracellular partners. LC-MS analysis showed that LOXL1 could interact with multiple proteins involved in the antiapoptotic pathway through complicated regulatory mechanisms. We found that LOXL1 targeted BAG2 and stabilized the BAG2 protein. In turn, BAG2 conferred glioma cell antiapoptotic activity to resist IR stress. The interaction between LOXL1 and BAG2 depends on LOXL1 enzymatic activity, suggesting that LOXL1 exerts its activity on the side chain of BAG2-K186.

The expression of LOX family genes is regulated by several signaling pathways. TGF-beta upregulates the expression of all genes in the LOX family [32]. After the induction of EMT by hypoxia, the expression of the *LOX* and *LOXL2* genes is mandatorily expressed by the transcription factor hypoxia-inducible factor [9]. Both the Ras/MAPK pathway and the PI3K/Akt pathway lead to the upregulation of VEGF and angiogenesis [6]. In the present study, under nonadherent conditions, the VEGFR/Src axis in glioma cells activates the transcription of the *LOXL1* gene by increasing the binding of CEBPA to its promoter region.

Finally, in clinical practice, the LOXL1 level in patient blood could indicate glioma progression, suggesting that LOXL1 acts not only as a potential target but also as a biomarker of gliomagenesis.

## Supplementary information

Supplementary figure legend

Supplementary Fig. 1

Supplementary Fig. 2

Supplementary Fig. 3

Supplementary Fig. 4

Supplementary Fig. 5

Supplementary Fig. 6

Supplementary Table. 1
