# Supplementary material for: LOXL1 confers antiapoptosis and promotes gliomagenesis through stabilizing BAG2
Source: Cell Death Differ. 2020 May 18;27(11):3021–36. doi: 10.1038/s41418-020-0558-4 (PMC7557908; doi:10.1038/s41418-020-0558-4)
Supplement: Supplementary file 1 — Supplementary figure legend [file 41418_2020_558_MOESM1_ESM.docx]

**Supplementary Fig. 1 a** Left panel, clinical significance of the LOX family from The Human Protein Atlas (https://www.proteinatlas.org); right panel, the prognostic analysis of LOXL1 was performed using four datasets (TCGA-395, TCGA-540, GSE16011 and GSE43378) from “R2: Genomics Analysis and Visualization Platform (<http://r2.amc.nl)>”. **b** The prognostic analysis of LOXL1 was performed using our own clinical tumor cohorts (n=80) by using log-rank test (up panel, *P* = 2e-5), and multivariate analysis (HR=3.079; 95% CI, 1.692-5.604; *P* = 2.3e-4 ) after controlling for age, gender and IDH mutation.. **c** mRNA expression levels of the LOX family from TCGA database. **d** CCK8 assay measures the proliferation of U87 cells overexpressing LOXL1 (the data are presented as the means ± SD, two-way ANOVA). **e** CCK8 assay measures the proliferation of LN18 cells after depletion of LOXL1 (the data are presented as the means ± SD, two-way ANOVA). **f** LOXL4 was silenced in LN18 cells and the proliferation (the data are presented as the means ± SD, two-way ANOVA) and colony formation of shNT or shLOXL4 cells (the data are presented means ± SD, unpaired t test, two-tailed) were analyzed. **g** Cell cycle analysis of U87-Vec and U87-LOXL1 cells or LN18-shNT and LN18-shLOXL1 cells cultured under 3D conditions.

**Supplementary Fig. 2** **a** H&E staining was performed to confirm tumor tissue in brain tumors composed of U87-LOXL1 cells or U87-Vec cells after IR treatment (means ± SD, unpaired t test, two-tailed). **b** Tumors were dissected from mice brain and stained with antibody against Ki67. Statistical analysis of positive Ki67 staining was shown on the right. (Scale bars: 50 μm). **c** Survival time after mice injected with glioma cells. The data are presented as the mean survival time ± SD of 4 mice per group.

**Supplementary Fig. 3** **a** Coomassie Brilliant Blue-stained SDS page gel showing the purification of LOXL1 from duplicate preparations of U87-LOXL1 cells. **b, c** Transient transfection of small hairpin RNAs knocked down BAG2 and impaired the invasion of LN18 and GSC11 cells. **d** The expression levels of LOXL1 and BAG2 are positively correlated (R=0.42) in glioma and the R value ranks the fourth in all the tumor types by using GEPIA, an interactive web server for analyzing the RNA sequencing expression data of 9,736 tumors and 8,587 normal samples from the TCGA and the GTEx projects.

**Supplementary Fig. 4** **a** The enzymatic activity of LOXL1 has no effect on mRNA level of BAG2. **b** The depletion of LOXL1 induced a slightly reduction of BAG2 mRNA level. **c** BAG2 protein level was maintained by LOXL1 in LN18 cells. shNT and shLOXL1 cells were treated with CHX (Cycloheximide, 1μM) in a time course. β-actin was used as an internal control.

**Supplementary Fig. 5** Mass analysis identified BAG2 K189 ubiquitin. The spectrum of ubiquitin-modified K189 peptide was identified with a m/z value of 384.90 and a SEQUEST XCorr value of 2.35.

**Supplementary Fig. 6** **a** Inhibition of TGF-beta signaling does not affect the up-regulation of LOXL1 in nonadherent cells. **b, c** The efficiency of the inhibitors used in Fig. 6**a** was verified using a specific antibody or luciferase assay (means ± SD, unpaired t test, two-tailed). **d** Inhibitor screen of central kinases required for the up-regulation of LOXL1 in GSC11 cells. **e** Knocking down of SRC gene reduced LOXL1 protein in GSC11 cells. **f** Src inhibitor reduced LOXL1 expression in LN18 cells (means ± SD, unpaired t test, two-tailed). **g** A ChIP assay identified that CEBPA targeted the LOXL1 promoter at 480 base pairs upstream of the TSS in GSC11 cells (means ± SD, unpaired t test, two-tailed). **h** Knocking down CEBPA reduced LOXL1 expression in GSC11 cells. Four pairs of siRNAs were applied to target the CEBPA gene. **i** Representative western blots showing CEBPA, LOXL1 and BAG2 levels in 14 glioma samples. β-actin was used as an internal control.

.
