# Supplementary figures and images for: LOXL1 confers antiapoptosis and promotes gliomagenesis through stabilizing BAG2
Source: Cell Death Differ. 2020 May 18;27(11):3021–36. doi: 10.1038/s41418-020-0558-4 (PMC7557908; doi:10.1038/s41418-020-0558-4)

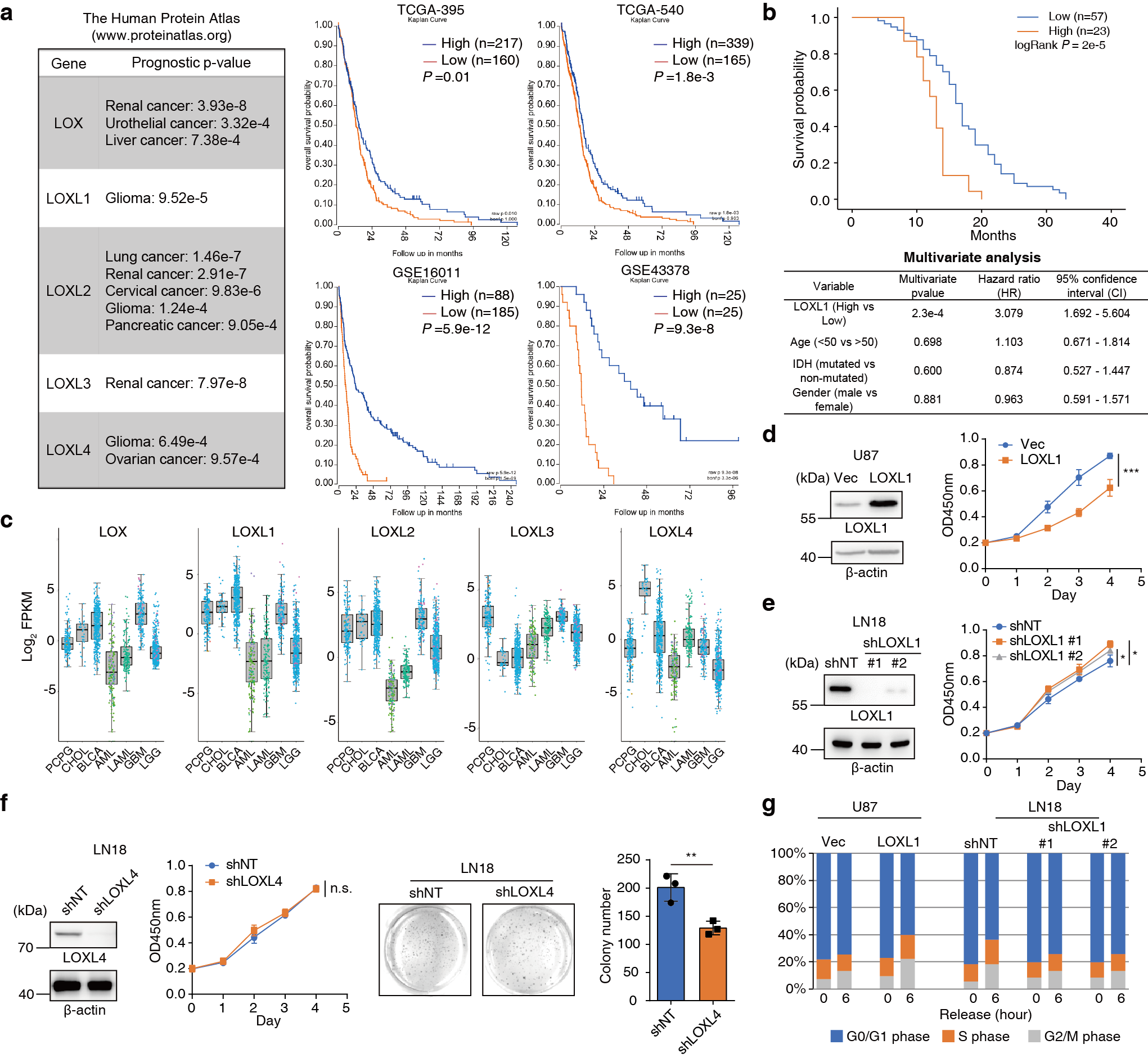

Supplement: Supplementary file 2 — Supplementary Fig. 1 [file 41418_2020_558_MOESM2_ESM.png]

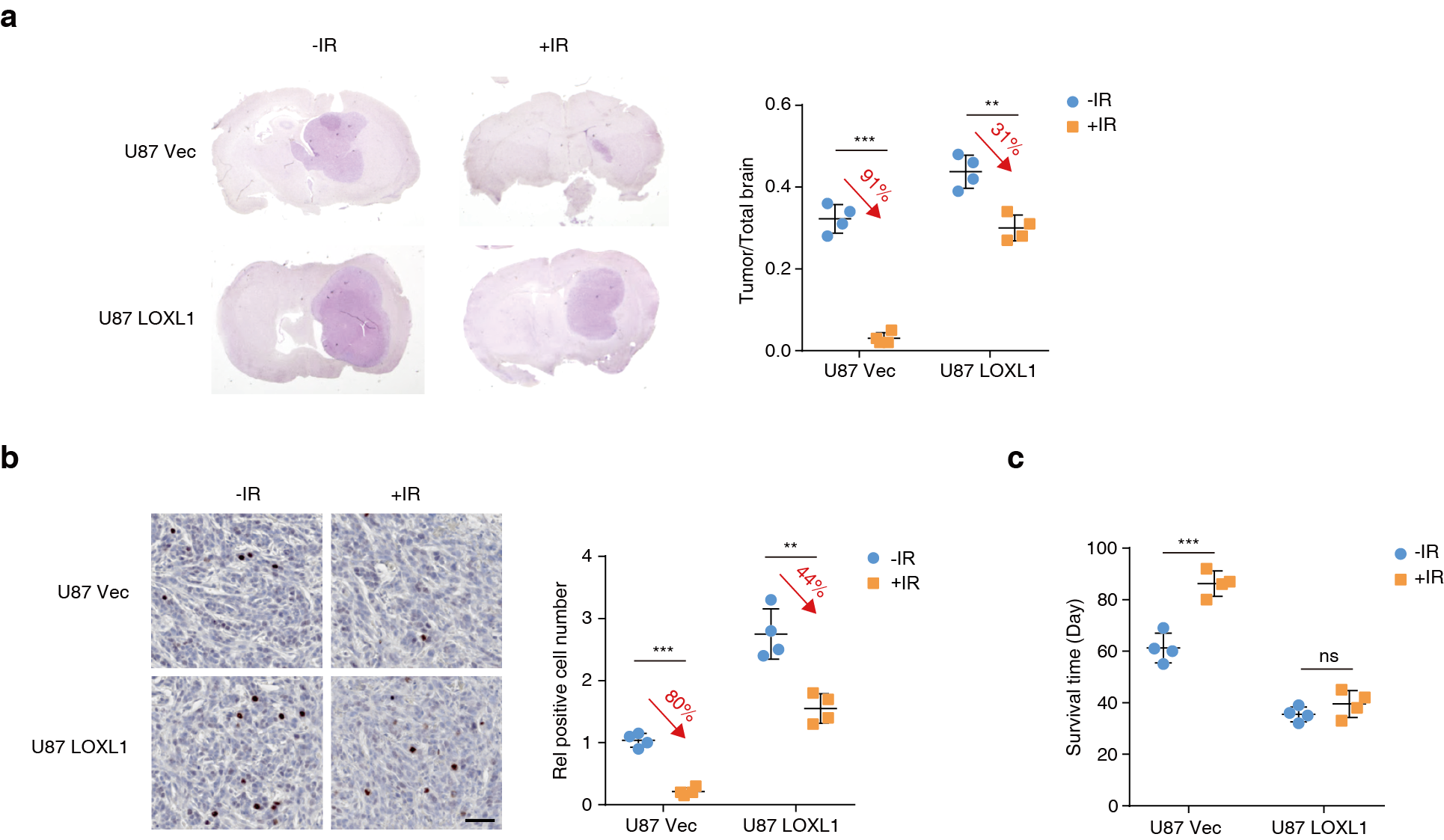

Supplement: Supplementary file 3 — Supplementary Fig. 2 [file 41418_2020_558_MOESM3_ESM.png]

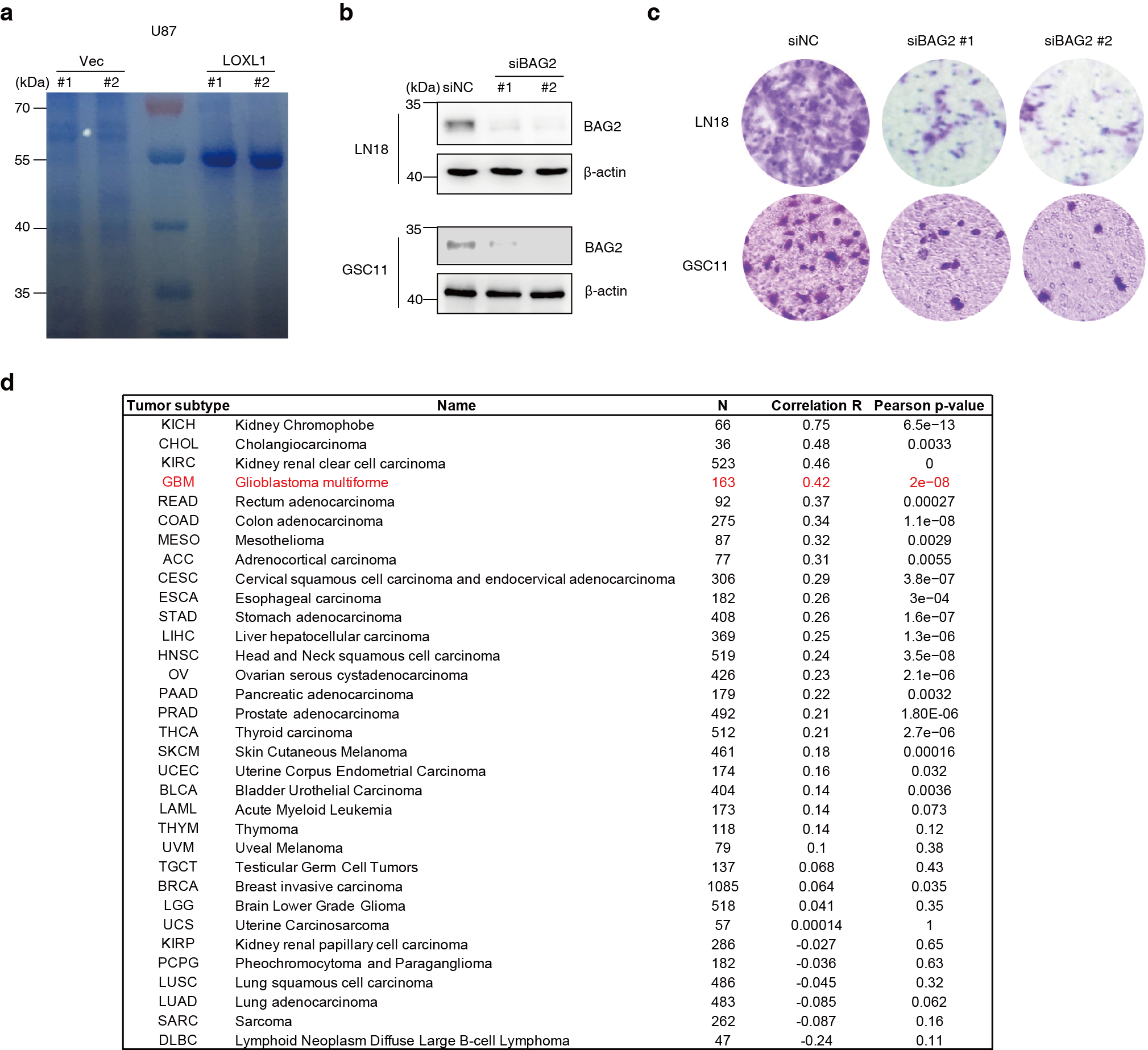

Supplement: Supplementary file 4 — Supplementary Fig. 3 [file 41418_2020_558_MOESM4_ESM.png]

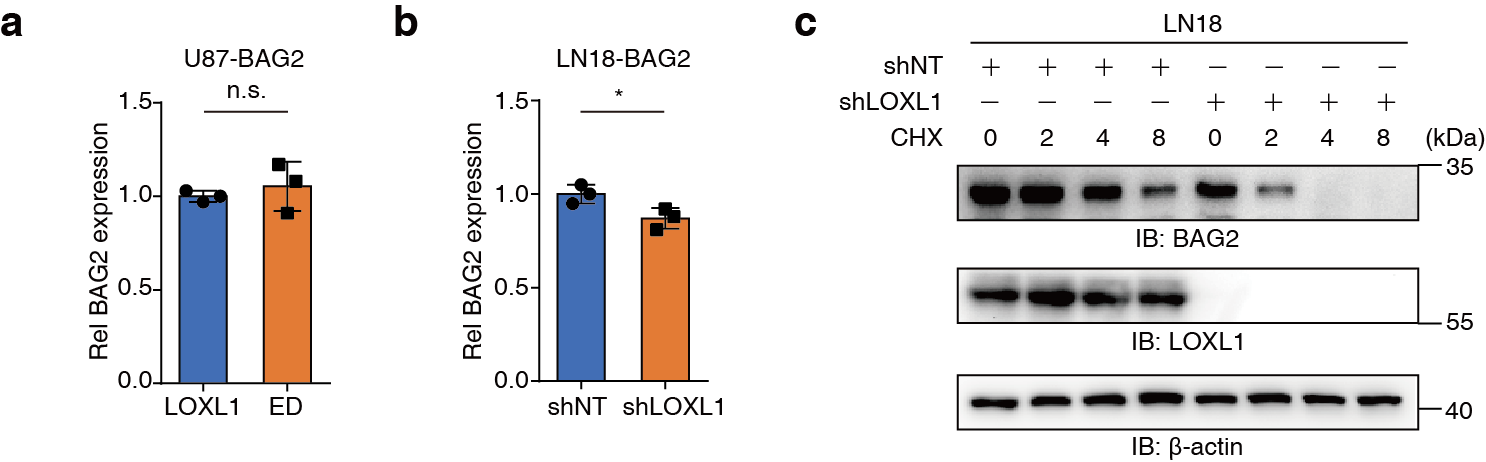

Supplement: Supplementary file 5 — Supplementary Fig. 4 [file 41418_2020_558_MOESM5_ESM.png]

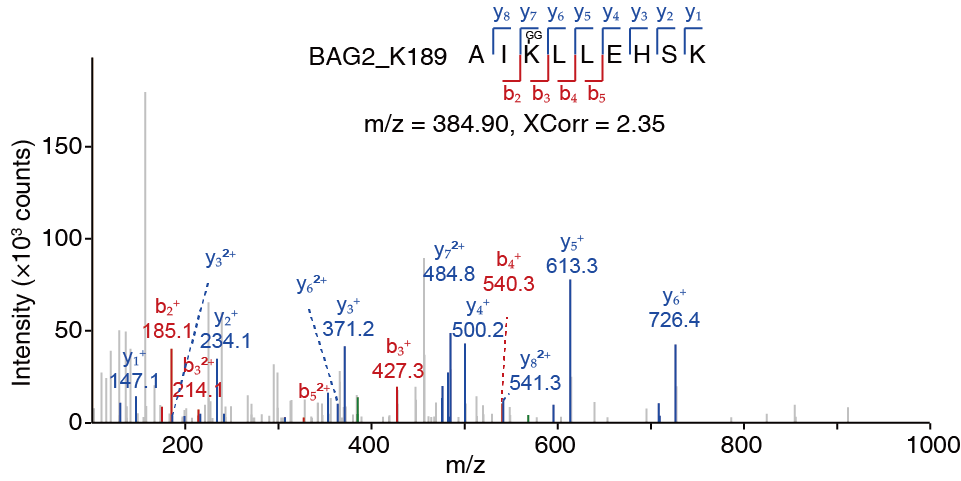

Supplement: Supplementary file 6 — Supplementary Fig. 5 [file 41418_2020_558_MOESM6_ESM.png]

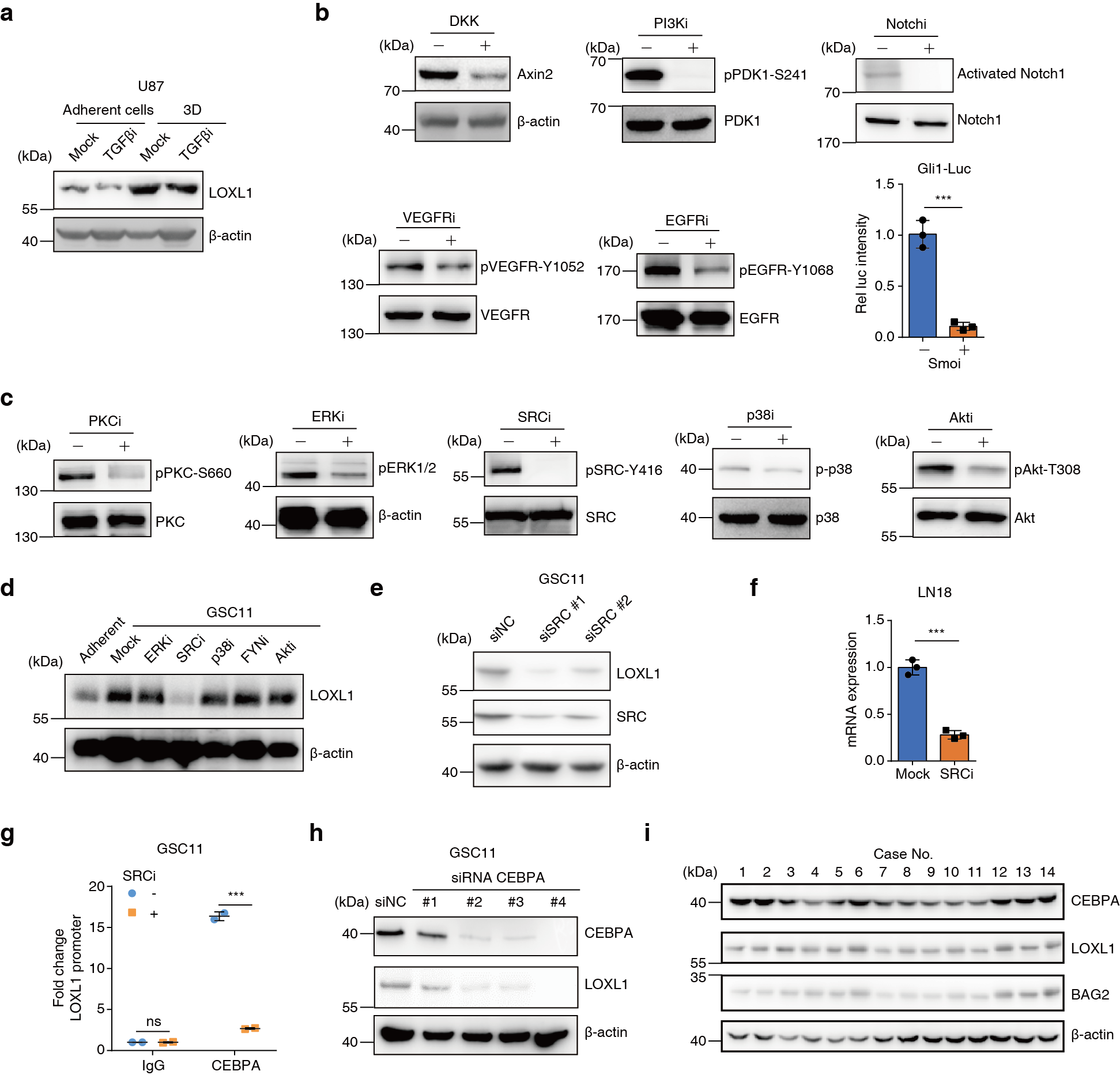

Supplement: Supplementary file 7 — Supplementary Fig. 6 [file 41418_2020_558_MOESM7_ESM.png]
